# Organic biopolymers of venus clams: Collagen-related matrix in the bivalve shells with crossed-lamellar ultrastructure

**DOI:** 10.1016/j.bbrep.2021.100939

**Published:** 2021-02-12

**Authors:** Oluwatoosin B.A. Agbaje, J. Gabriel Dominguez, Dorrit E. Jacob

**Affiliations:** a Department of Earth and Environmental Sciences, Macquarie University, Sydney, Australia; b Department of Biological Sciences, Macquarie University, Sydney, Australia

**Keywords:** Polysaccharides, Chitin, Collagen, Glycosaminoglycans, Biomineralization, ATR-FTIR

## Abstract

**Background:**

Biochemical studies and spectroscopic techniques have shown that chitin-silk fibroins are common in nacroprismatic bivalve shells. However, the nature of organic biopolymers in the less well studied shell architectures, such as crossed lamellar shells, remain unknown. Here, two venus shells, *Callista disrupta* and *Callista kingii*, with crossed lamellar ultrastructure have been studied.

**Methods:**

We employed thermal gravimetric analysis, optical-, confocal- and scanning electron-microscopes, gel-sodium dodecyl sulfate (gel-SDS), FTIR, ultra-performance liquid chromatography and high-performance anion-exchange chromatography system with pulsed amperometric detection to analyse organic macromolecules in *the* shells.

**Results:**

Thermal analysis showed a low concentration of organic macromolecules in *C. disrupta* (1.38 wt%) and in *C. kingii* (1.71 wt%). A combination of biochemical protocols, including Calcofluor White staining and FTIR spectroscopic assessment, indicate that amino-polysaccharide chitin together with proteins, are present in the organic scaffolding of *the* shells. Scanning electron microscope of insoluble acid biopolymer extracts as well as FTIR technique show that the hierarchical structural organizations of organic biopolymers consist collagen-related matrix. Our histochemical fixing and staining techniques reveal many discrete proteins and glycoproteins from soluble organic macromolecules on the gel-SDS. We show here ‘singlet’ and ‘doublet’ glycosaminoglycan bands that are far above 260 kDa.

**General significance/conclusions:**

The presence of collagen matrix in *Callista* shells shows promise for the new source of biomaterials. Most importantly, the structural organization of the proteinaceous motif is predominantly helical structures and not silk-fibroin unlike in nacreous bivalve shells.

## 1 Introduction

Biomineralized organisms produce various organic-inorganic nanocomposite minerals, namely calcium carbonate, calcium phosphates and silica [1], whose mechanical properties are often outstanding. Organic biomolecules composed of lipids, acidic proteins, glycoproteins and polysaccharides can be components of bivalve shells and are widely known to be part of different shell architectures [[2], [3], [4]] and form a range of different organic structures, such as fibrous, helical, layered, tubular, and intermediate structures [5,6].

The organic biomacromolecules within calcareous bivalve shells are present in small concentration, <5 wt% [7], and have been shown to control the nucleation and crystallization of the carbonate across all hierarchies [8,9]. The majority of studies up to now concentrated on the nacroprismatic ultrastructure (e.g. Refs. [4,7,[10], [11], [12], [13], [14]]), and less so on the many other bivalve shell structures that exist in nature [10,[15], [16], [17], [18], [19], [20]].

Generally, the organic macromolecules in nacroprismatic shells consist of water-soluble and insoluble portions. The soluble moiety is relatively low in content, <2 wt%, and is rich in proteins with acidic side chains and glycoproteins (e.g. Refs. [4,19]). The insoluble organic moiety is made up of hydrophobic constituents, such as chitin, a homopolymer of *N*-acetyl-d-glucosamine in its *β*-conformation [1,21] and silk fibroins [7,10,11]. Both components together form multi-scale chitin-protein scaffolding structures which are intimately intergrown with the inorganic matrix of the shells and influence crystal nucleation and growth (e.g. Ref. [9]). These observations have led to the general view that the organic structure in non-nacreous shell architectures probably consists of polysaccharide chitin fibres [22] coated with soluble organic macromolecules, that is, rich in aspartic and glutamic acids.

Previous studies have supported this hypothesis (or composition), in part, and revealed polysaccharide chitin in homogeneous [10] and crossed lamellar ultrastructures [15]. In contrast, soluble protein biomolecules are variable in different shell types. While silk fibroin and its derivatives are common in nacroprismatic shells [7,9,11,14], the organic compositions in homogeneous and crossed lamellar shells are related to collagen-like matrix [10,19]. In addition, the roles of other extracellular matrices and glycosaminoglycans, which have been identified in shells [10,23] in the formation and the material properties of the biominerals are yet to be clarified.

In this study, we chose bivalve venus shells (*Callista disrupta* and *Callista kingii*) to further understand the organic components of bivalve shells with crossed lamellar architecture. The crossed lamellar ultrastructure is the most common molluscan ultrastructure but has only been receiving increased attention lately (e.g. Refs. [15,17,19,24]). This ultrastructure consists of several orders of differently-sized lamellae enveloped in organic phases, which can be grouped into platelet-like or fibre-like matrices [15,25]. We present here the functional biomolecules in the shells of *Callista* spp.

### 1.1 Architecture of *Callista* bivalve shells

**Fig. 1:**
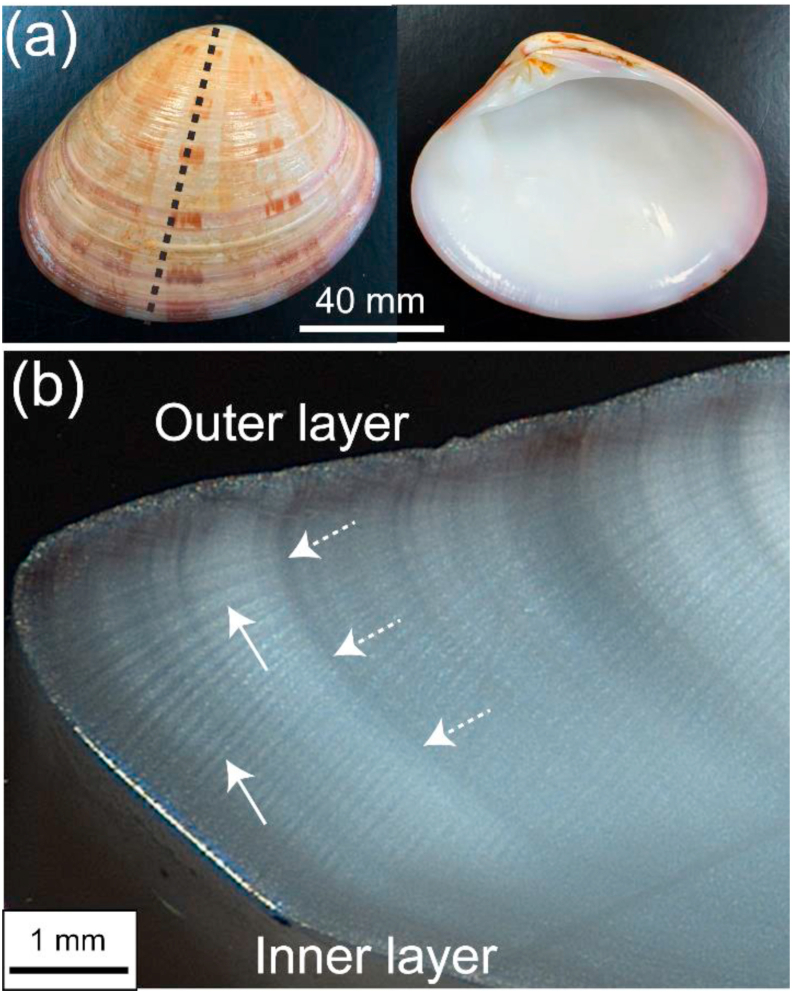
(a) Shell of *Callista* with the dashed line showing where the shell was sectioned. (b) Optical micrograph of the radial cross-section at the ventral marginal. The arrows depict the striped pattern and dashed arrows show growth line.

*Callista* shells (Fig. 1a) comprise an outer shell layer and an inner shell layer (Fig. 1b) that are both made of aragonite. Growth lines transect the structure and represent time periods of physiologically reduced shell growth [26]. Depending in the species, growth lines can be more or less mineralized and in *Callista* shells, they are organic-rich layers consisting of varying contents of organic constituents. The striped patterns seen in Fig. 1b (arrow) are caused by the formation of inorganic matrix and correspond to the irregular shaped first order prisms [25], each of them being composed of smaller bundles of elongated units, i.e. second order prisms, that are stacked up diagonally along the stripes (Fig. 1b). The striped pattern in the inner layer is not prominent probably due to the non-mineralized components. In contrast, a characteristic striped pattern in the outer layer is angled at ~90° to the growth lines of the inner layer.

## 2 Material and methods

### 2.1 Material

Shells of recently alivemarine bivalves: *Callista disrupta* (Veneridae; Sowerby, 1853) and *Callista kingii* (Veneridae; Grey in King, 1827) were used for this study (Fig. 1).

**Field Emission Gun-Scanning Electron Microscopy (FEG-SEM) and Confocal Microscopy.***Callista* shells were broken into few millimetre-sized fragments and were then etched with 1% EDTA disodium salt (wt/vol). The etched samples and the lyophilized acid insoluble organic extracts were gold-coated and imaged with a JEOL JSM-7100F FEG-SEM at Macquarie University. A fluorescence microscopical investigation of the shells was performed using Calcofluor White M2R, CFW (Sigmal Aldrich; Fluorescent Brightener F-3543; 0.1%) under an Olympus Fluoview FV1000 laser confocal microscope (Olympus, Japan) with a laser diode (405 nm) and a UV-filter for the 425–525 nm range. Prior to histochemical staining, samples were decalcified with 10% glacial acetic acid at 4 °C for 1 week.

**Shell preparation and organic matrix extraction.** The thin outermost organic layer of the *Callista* shells (periostracum) was scraped off with a scalpel. After that, the entire shells were submerged in hydrogen peroxide (30%; Merck KGaA, 64 271 Darmstadt; Germany) for 2 h combined with ultrasonic treatment to remove any remaining surface absorbed organic contaminants, washed with Milli-Q water and then air dried at room temperature. Shell samples were divided into two portions of which one portion was powdered with a rock crusher. To obtain the water-soluble organic moiety (SOM) and acid soluble moiety (ASM) a portion of the powdered sample was decalcified in 1 mM HCl and 0.1 N trichloroacetic acid plus phosphate buffer (pH 7.4) at room temperature [15]. For the second portion, large shell pieces were demineralized in strong hydrochloric acid as described in the previous work [10]. Milli-Q water was added intermittently to reduce excessive frothing and the recovered shell biopolymer after this is called acid insoluble organic extracts (IOE). Subsequent preparation involved bleaching in 35% hydrogen peroxide and 5% sodium hydroxide (2:1) for ~45 min to further de-proteinize and/or remove pigments. Samples were then washed in Milli-Q water until pH of ~6.8 was obtained, the organic extracts were freeze-dried and weighed. Commercially available type IV collagen, extracted from human placenta (Sigma-Aldrich; C7521) and chitosan extracted from shrimp shells (Sigma-Aldrich; C3646) were used as standards.

**Characterization of the organic matrix.** Fourier Transform Infrared (FTIR) spectra were acquired from lyophilized samples at a 2 cm^−1^ resolution with 64 accumulations in the range 4000–600 cm^−1^, using a Thermo Nicolet iS10 FTIR spectrometer (Nicolet, MA, USA) equipped with an attenuated total reflection device. Each sample spectrum was corrected for a background spectrum collected prior to each sample measurement.

Thermal gravimetric analysis (TGA) and differential thermal analysis (DTA) were carried out on shell powders using a TGA 2050 thermogravimetric analyser (TA Instruments, USA). The samples (ca 30 mg) were heated at a linear gradient of 10 °C/min from 21 °C to 1000 °C.

Saccharide compositions of water-soluble organic and acid soluble moieties were quantified following the method described in the previous work [15]. Each lyophilisate was hydrolysed in 2 M trifluoroacetic acid at 100 °C (4 h) and 8 M HCl at 100 °C (6 h), respectively. The sugar contents of the hydrolysate samples (glucosamine, galactosamine, mannose, xylose, fucose, galactose and glucose) were determined on a high-performance anion-exchange chromatography system with pulsed amperometric detection (HPAEC-PAD) fitted with a BioLC amino trap guard column (3 × 50 mm) connected to a CarboPac PA10 column (4 × 250 mm) (Dionex Corp., Sunnyvale, CA, USA) held at 25 °C and eluted by using sodium hydroxide at a flow rate of 0.5 mL/min. The sugar contents expressed as an ng/mg, represent the average of duplicate results.

For amino acid analysis, aliquots of the shells extracts were acid-hydrolysed in 6 N HCl at 110 °C for 24 h under nitrogen atmosphere. Ensuing evaporation of the solution to dryness, the resulting hydrolysates were analysed using an ACQUITY ultra-performance liquid chromatography (UPLC) system and BEH RP C18 1.7 μm column (Waters Corporation, Milford, MA).

#### 2.1.1 Determination of matrix proteins and chitin derivatives on polyacrylamide gel

The separation of shell matrix constituents of both, the water-soluble organic moiety and acid soluble moiety, was performed under denatured conditions with Laemmli sample buffer containing dithiothreitol. Equal amounts of 100 μg shell matrix were run by electrophoresis in a pre-cast 4–12% NuPAGE® Bis-Tris gel according to protocols supplied by the manufacturer (Invitrogen; Carlsbad, CA, USA). Novex® Sharp Pre-Stained Protein Standard (Invitrogen; 5 μL) was used as a size marker. After electrophoresis, gels were stained for proteins with silver nitrate and for potential glycosylation with Alcian Blue [7,15]. In brief, water-soluble and acid soluble extracts were stained with Alcian Blue 8GX at pH 1, and subsequently stained with silver nitrate. Chitin deacetylated (CDA) activity was analysed after sodium dodecyl sulfate–polyacrylamide gel electrophoresis (SDS-PAGE) using the method described [15].

## 3 Results

### 3.1 Histochemical localization of shell biopolymers and thermal gravimetric analysis

**Fig. 2:**
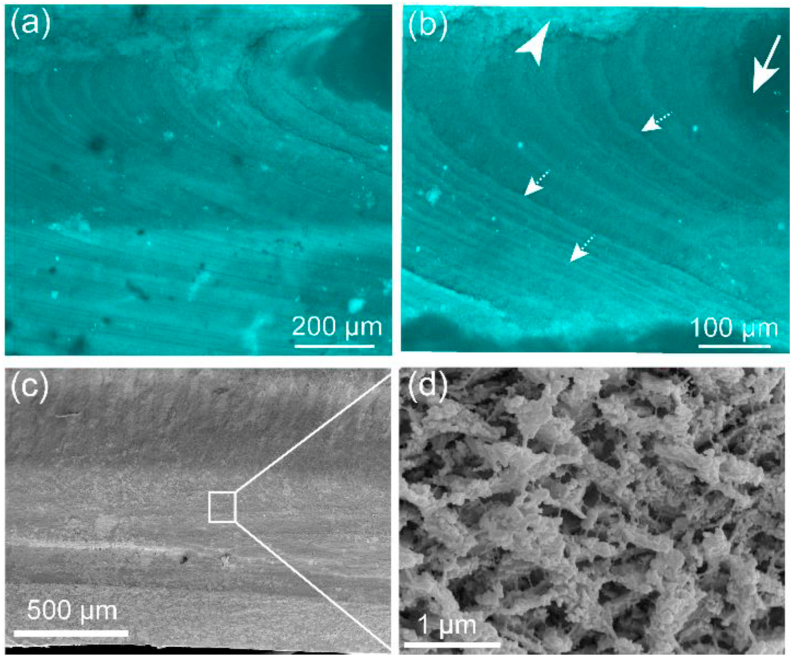
(a) Confocal microscope image showing distribution of polysaccharide-based biopolymers within the growth bands stained with Calcofluor White M2R (b) Higher magnification of inner portion of the outer shell layer. The white arrowhead point towards outer portion of the outer shell layer. The white arrow defines the outside surface of the shell. (c, d) Field emission gun scanning electron micrographs of *the* shell after etching showing ultrastructures and organic fibres. Higher magnification reveals rod-type crossed lamellar parallel to the co-marginal plane.

After etching, the organic matrix within the growth lines of *Callista* shells are well stained with Calcoflour White M2R, CFW (Fig. 2a and b; SI Fig. 1). The shell biopolymers are visualized using the fluorescent marker CFW which forms hydrogen bonds with *β*-(1–3)- and *β*-(1–4)-linked polysaccharides and shows a strong affinity for carbohydrates while proteins are not highlighted [15,27]. Also, SEM imaging reveals distinct organic fibre within the inorganic matrix (Fig. 2c and d).

**Fig. 3:**
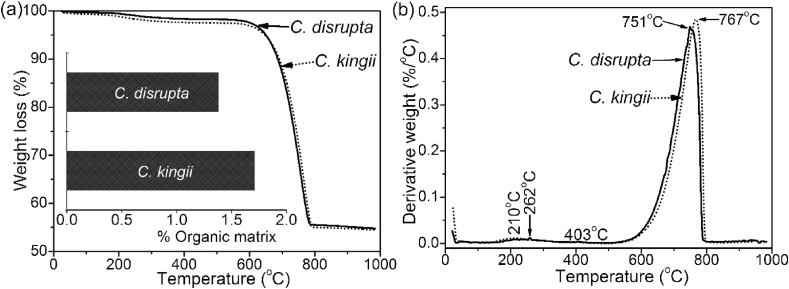
Thermal gravimetric analysis (TGA; a) and differential thermal analysis (DTA; b) for a linear gradient from 21 °C to 1000 °C. The range 150–500 °C was used for calculation of the bioorganic contents. Total amounts of organic matrix in *Callista disrupta* and *Callista kingii* are 1.38 wt% and 1.71 wt% (insert in a). The broad peaks at around 210 °C and at 262 °C are due to the release of organic biomolecules, the peak at 403 °C is caused by the transformation of aragonite to calcite. The peaks at 751 °C and 767 °C are due to the decomposition of calcium carbonate and release of CO_2_.

Thermal gravimetric analysis was used to determine the total amount of organic content in the shells (excluding the periostracum). The total weight loss of organic contents of 1.38 wt% for *C. disrupta* and 1.71 wt% for *C. kingii* occurs in the 150–500 °C range and is due to the decomposition of the organic matrix (Fig. 3a). This agrees well with the amount of shell biopolymers in other venus shells [20] and other molluscan shells with crossed lamellar architecture [15]. The differential thermal analysis of shell samples exhibits multistage decompositional steps (Fig. 3b). The first occurs in the 210–403 °C range is attributed to the combustion of the complex mixture of proteins, glycoproteins and polysaccharides occluded in the shell biominerals as well as the transformation from aragonite to calcite. This is followed by a breakdown of calcium carbonate to calcium oxide at 751 °C for *C. disrupta* and 767 °C wt% for *C. kingii*. The weight loss of 43 wt% (Fig. 3a) for both shell biominerals at this temperature is in accordance with the theoretical value of calcium oxide (44 wt%) released from the calcium carbonate decomposition.

### 3.2 Bulk composition of shell macromolecules

**Fig. 4:**
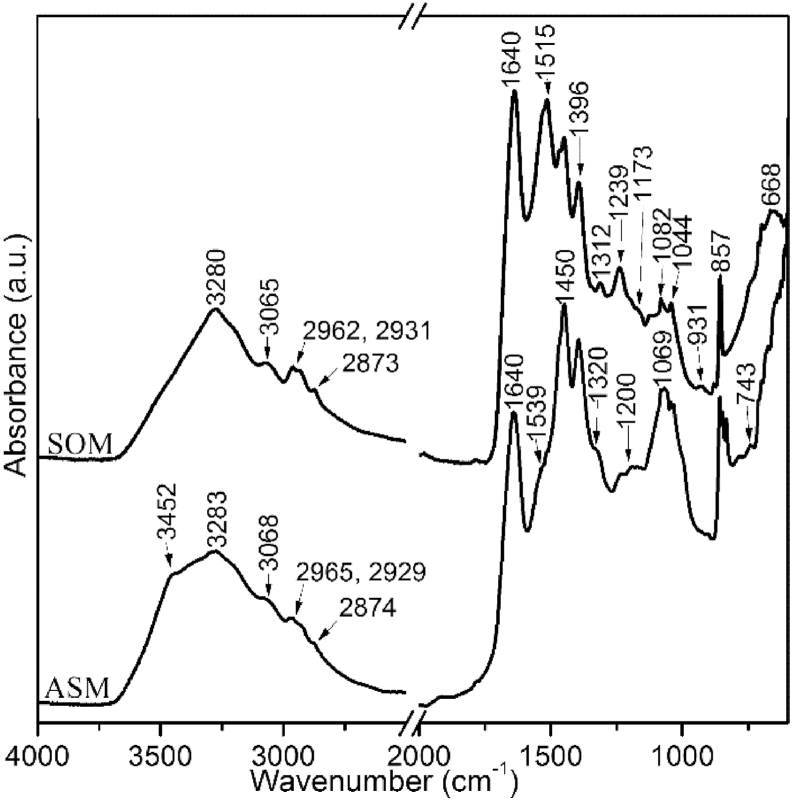
FTIR spectra of Water-Soluble Organic Moiety (SOM) and Acid Soluble Moiety (ASM). The extracts show characteristic absorptions of *β*-sheet and/or random coiled form at 1640 cm^−1^ for amide I and at ~1239 cm^−1^ for amide III. See Table 1 for band assignments.

The functional properties of the lyophilized water-soluble organic moiety and acid soluble moiety are summarized in Table 1. In both extracts, the FTIR spectra are characterised by the occurrence of amide A, B, I, II and III. Bands at around 3272 cm^−1^, 3065 cm^−1^ and between 2962 cm^−1^ and 2873 cm^−1^ assign to the N–H bound of amide A, B and C–H stretching groups, respectively. Also, a strong band at 1640 cm^−1^ (Fig. 4) is attributed to the *β*-sheet or random coiled form of amide I C=O bond. Different patterns occur in the spectra of water-soluble organic moiety and acid soluble moiety. A well-defined amide II band at 1515 cm^−1^ (tyrosine) and a small amide III band at 1312 cm^−1^ (C–N and N–H bound) for water-soluble organic moiety show small shoulders at around 1539 cm^−1^ (C–N bound) and 1320 cm^−1^ in the spectrum of acid soluble moiety. An 833 cm^−1^ band is only visible in the spectrum of acid soluble matrix. Also, the carbohydrate band at 1069 cm^−1^ is more prominent in the acid soluble moiety extract and results from the large proportion of monosaccharides (Table 2). This band may point to the presence of sulphated glycosaminoglycans, SO_3^−^_ vibration [7,11]. A band at ~1174 cm^−1^ is correlated with proteoglycan protein for both extracts. Strong absorption bands at ~1450 cm^−1^ and 1395 cm^−1^ assign to the asymmetric bending of CH_3_ vibration and carboxylate absorption bands, respectively [7,11,[28], [29], [30], [31]], see Table 1. Since sulfate is known to produce bands in the 1250-1200 cm^−1^ range in nacreous shells [11], the band at 1239 cm^−1^ (and 1234 cm^−1^) is interpreted as a sulfate band. In addition, we identify weak collagen-related bands at ~1201 cm^−1^ and 1282 cm^−1^ in the amide III region (Table 1, Fig. 4). The bands at 1239 cm^−1^ and 1234 cm^−1^ can also be the indicative of *β*-sheet and/or random coils of collagen in the amide III region [28,32]. The fingerprint region in the 750-600 cm^−1^ range and a 791 cm^−1^ band for the samples studied are attributed to the polysaccharide moieties, probably *N*-acetylglucosamine [7,31], see Table 1.

**Table 1:** Position and assignment of the FTIR bands of shell macromolecules, untreated type IV collagen and untreated chitosan in the 4000-600 cm^−1^ range.

SOM	ASM	IOE	Type IV collagen	Chitosan	Assignment
	3452			3360	OH stretching
3280	3283	3274	3292	3291	NH asymmetric stretching (Amide A)
3065	3068	3078	3087	3095	NH symmetric stretching + C–H aromatic stretching
2962	2965	2962	2960		CH_3_ asymmetric stretching
2931	2929	2932	2929	2916	CH_2_ asymmetric stretching
2873	2874	2876	2878	2874	CH_3_ symmetric stretching
		2854	2852		CH_2_ symmetric stretching
1640	1640	1635	1634	1652	C=O stretching (Amide I)
				1592	C=O stretching
1532*	1539	1532	1549	1562	N–H bending + C–N stretching (Amide II)
1515		1515			C=C stretching of tyrosine
1469	1469*				C–O–C bending vibration COO^−^
1452	1450	1449	1452	1454*	CH_3_ asymmetric bending, CH_2_ stretching
1396	1395	1398	1398	1422	COO^−^ symmetric stretching
				1375	C–CH3 rocking
		1337	1338		CH_2_ wagging (Collagen integrity)
1312	1320	1317	1317	1315	N–H in-plane bending + C–N stretching
1282*	1282*	1280*	1280		CH_3_CH_2_ wagging of glycine
				1260	C–H stretching, C=O rocking
1239	1234	1236	1236		CH_2_ wagging, C–N stretch/SO_3^−^_ asymmetric stretching (Amide III)
1202	1200	1203*	1203	1201	CH_2_ wagging from glycine backbone and from proline sidechain overlap with C–O deformation of carbohydrate
1173	1174*	1175			C–O–C stretching
			1151		C–O–C stretching of carbohydrate
1124		1131			C–O–S asymmetric stretching
		1115	1117		C–O asymmetric stretching
1082	1069	1084	1078		C–O stretching of the carbohydrate
					/SO_3^−^_ symmetric stretching
1044	1044	1042*		1061	C–O–H stretching of the carbohydrate
		1031	1030	1028	C–O stretching of the carbohydrate
998		993*		995	C–O–H deformation of carbohydrate
				951	CH_3_ deformation
931		939	939		C–C stretching mode/SO_3^−^_ stretching
			921		C–C stretching mode
		891*		895	CHx deformation, C–O–C glycosidic bond
881		877*	880		CH_2_ rocking
857	857, 845	851	852		C–C stretching, C–H bending/SO_3^−^_ stretching
	833	831			C–C stretching, C–H bending
		807	805		C–O stretching of collagen crosslink
	791 743	746	762	754*	C–H out-of-plane bending
					C–H out-of-plane bending
		701	702	710	C–H out-of-plane bending
668				668	C–H out-of-plane bending

SOM and ASM represent water-soluble organic moiety and acid soluble organic moiety, respectively. See extraction methods for the details of acid insoluble organic extracts (IOE). * = shoulder. See material and methods section for the extraction methods.

**Table 2:** The composition of the organic moieties of *Callista* shells. (a) Amino acid composition (mole %) of the water-soluble organic moiety (SOM) and of the acid soluble moiety (ASM). (b) Monosaccharide composition of SOM and of ASM extracts. The values are expressed in μg/g and in wt%. TR, trace.

(a) Percentage of the total amino acid		
Amino Acid	SOM	ASM
His^a^	0.2	0.4
Ser^b^	6.2	6.0
Arg^a^	6.2	4.4
Gly	13.3	11.8
Asx^a^ (Asp + Asn)	10.2	17.5
Glx^a^ (Glu + Gln)	7.5	10.6
Thr^b^	4.7	5.4
Ala^c^	7.3	6.3
Pro^c^	11.7	10.2
Lys^a^	3.3	4.0
Tyr^b^	2.7	2.6
Met^c^	1.4	0.3
Val^c^	6.1	5.5
Ile^c^	6.1	4.9
Leu^c^	7.5	5.2
Phe^c^	5.5	4.7
(b) Monosaccharide composition [μg/g of matrix (% of the total matrix composition)]		
Galactosamine	460 (26.1)	643 (16.8)
Glucosamine	327 (18.6)	528 (13.8)
Fucose	TR	TR
Glucose	534 (30.3)	1163 (30.4)
Xylose	219 (12.4)	939 (24.5)
Mannose	220 (12.5)	555 (14.5)
Total	1760 (100)	3828 (100)
Total Sugars	0.17%	0.38%

NB: ^a^ charged amino acid; ^b^ uncharged, polar amino acid; ^c^ hydrophobic amino acid; SOM: Water-Soluble Organic Moiety; ASM: Acid soluble Moiety. See material and methods section for the extraction methods.

### 3.3 Amino acid composition

The amino acid composition (in mol%) of the water-soluble organic and acid soluble moieties are presented in Table 2a. In the water-soluble organic extract, glycine, proline and aspartate account for 35.2% of the total amino acids, followed by glutamate, leucine and alanine, making up 22.3%. Contrarily, acid soluble organic moiety extract contains prominent amounts of aspartate (17.5%), followed by glycine, glutamate and proline. The three residues constitute 32.6% of the total amount of the amino acid.

In the acid soluble moiety, polar amino acid (aspartate and glutamate) is significantly higher (28.1%) compared to the water-soluble organic moiety (17.7%). Threonine, valine, alanine, leucine and isoleucine in the acid soluble moiety comprised 27.3% of the total amino acids while arginine, phenylalanine, valine and isoleucine (23.9% of the total amino acids) are fairly distributed in water-soluble organic moiety. Both extracts contain serine at approximately 6%. The lowest amount of histidine in the water-soluble organic moiety (0.2%) is similar to that of the acid soluble moiety (0.4%), see Table 2a.

### 3.4 Monosaccharide composition

The water-soluble and acid soluble organic matrices exhibit neutral and amino sugars (Table 2b). In the soluble organic moiety, galactosamine and glucose are the prominent constituents that comprise 56.4% of the total amount of the sugar contents followed by glucosamine, mannose and xylose. The sum of the latter constituents represents 43.5% of the total amount of sugar contents. In contrast, glucose and xylose are the prominent sugar contents in acid soluble moiety. The two residues represent 54.9% of the total amount of the sugars, followed by galactosamine, mannose and glucosamine (45.1% of the total amount of sugars). The two extracts exhibit only traces of fucose. The total amount of neutral and amino sugars in each matrix represents 0.18 and 0.38 wt% for water-soluble organic moiety and acid soluble moiety. However, due to the method of extraction, particularly for the acid soluble moiety (see methods of extraction), the percentage of the sugar contents does not represent the totality of the carbohydrate in *Callista* spp. After the extraction of the organic macromolecules from the insoluble fraction by using trichloroacetic acid-phosphate buffer solution, we observed an insoluble fraction that may still contain a considerable amount of carbohydrates. While it is most likely that the percentages are underestimated, the SDS-PAGE staining method applied here allows us to detect chitin derivatives more accurately.

### 3.5 Separation of shell macromolecules on SDS-PAGE

**Fig. 5:**
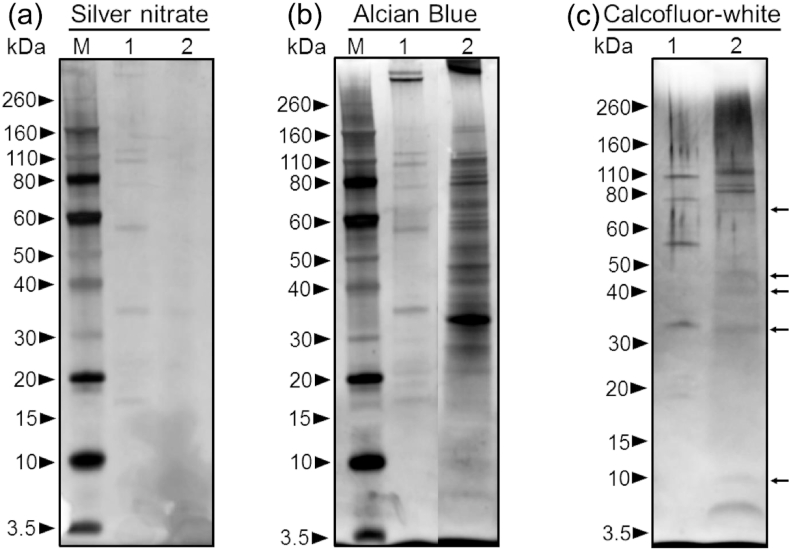
SDS-PAGE of water-soluble organic moiety (Lane 1) and acid soluble moiety (Lane 2) extracts. Lane M: Standard marker with masses in kDa. For Alcian Blue and Calcofluor White, see methods for detail. Arrows depict apparent molecular weights at around 70, 45, 40, 36 and 10 kDa. Bands far above 260 kDa (in a and b) point to glycosaminoglycans and/or proteoglycans band. In (b), lane 1 shows doublet bands far above 260 kDa. NB: Standard marker shows negative staining with Calcofluor White dye.

A sequential soluble organic fraction of *Callista* shells are similarly characterised by mono-dimensional gels (Fig. 5). Using Alcian Blue and/or silver nitrate, the total mixture of organic biomolecules in the water-soluble organic moiety contain many shell proteins that separated in SDS-gels: bands at around 18, 36, 58, below and above 110, and far above 260 kDa are visualized to stain with silver nitrate (Fig. 5a; lane 1). In addition to the bands mentioned above, doublet bands stained far above 260 kDa and additional bands stained faintly at apparent molecular weight 78 kDa (Fig. 5b; lane 1) with the Alcian Blue protocol (see method). The staining intensity of Alcian Blue is relatively higher than that of silver nitrate (Fig. 5a; lane 1). In contrast, the acid soluble moiety extract depicts a strong negative staining with silver nitrate (Fig. 5a; lane 2). In this case, the same fixation method was used, the gel was stained with Alcian Blue and was then enhanced with silver nitrate. After this treatment, the gel exhibited a number of prominent protein bands and a higher molecular mass far above 260 kDa was identified (Fig. 5b; lane 2). Our observations show that only silver nitrate, as opposed to combined staining, does not bind to these glycosylated acidic proteins.

Amino polysaccharides i.e. deacetylated chitin (or chitosan) are also investigated by SDS-PAGE (Fig. 5c). The water-soluble organic moiety is characterised by the presence of apparent molecular masses of 110, 78, 58 and 36 kDa (Fig. 5c; lane 1). These bands reveal that the water-soluble organic moiety consists of acidic glycoproteins as they stain in all SDS-gels (Fig. 5) — acidic glycoproteins are covalently linked with the sugar moiety. Also, the acid soluble extract demonstrates major molecular masses at 110 and 80 kDa, bands at around 5, 10, 35, 40, 45, 70 kDa and additional faintly stained bands (including bands above 80 and 110 kDa) are observed (Fig. 5c; lane 2).

Polysaccharide-based biomolecules in calcareous shells are generally modified by the insoluble proteins that often act as a framework and other biopolymers such as glycosaminoglycans [[3], [7], [10], [11], [12], [33]]. It is very challenging to isolate protein-free polysaccharide from calcareous biominerals as it is most commonly linked covalently with the shell-associated proteins [[10], [12]]. However, it was established that the CFW binds directly with sugar derivatives [34] and not with the proteins even when staining glycoprotein [35]. In this study, most of the prominent glycoprotein bands in Fig. 5b are indeed not visible/prominent with Calcoflour staining (Fig. 5c). For instance, glycosaminoglycans bands at far above 260 kDa are not visible. Besides, apparent molecular weights that show negative staining at around 5 and 10 kDa in Fig. 5b birefringent with fluorescence method (Fig. 5c).

## 4 Discussion

The intriguing complexity of most mineralized structures is a result of the interactions between organic macromolecules and the inorganic matrix. The shells of *Callista* spp. are highly mineralized with only small amounts of organic macromolecules, 1.71 wt% for *C. kingii* and 1.38 wt% for *C. disrupta*, compared to 1.4 wt% found in the compound composite prismatic and 2.2 wt% in crossed acicular ultrastructures [20] but unlike ~4 wt% found in nacre [7,36]. It is essential to note that the organic biopolymers in nacre consist of unusual acidic shell proteins [4,11,19], while organic macromolecules extracted from other shell architectures like crossed lamellar and homogeneous architectures have to date not been found to be aspartic-rich [15,18,19].

In the present study, total amino acid compositions and proportions from combined water-soluble organic moiety and acid soluble moiety investigation exhibit relatively higher amounts of aspartate (asparagine and aspartic acid), proline, serine and threonine (SI Fig. 2a). These residues are commonly known as glycosylation linkers in glycoproteins and associated with a relatively large amount of sugar-derivatives, probably proteoglycans [33]. Previous work showed that these aggregates are involved in biomineral nucleation and crystal growth [37]. Shell macromolecules of the samples studied here are weakly glycosylated, with total sugar contents <1 wt% (Table 2b), our results identify well-defined glycoprotein bands. A ‘doublet’ (SOM, lane 1) and ‘singlet’ (ASM, lane 2) bands far above 260 kDa (Fig. 5) are attributed to the glycosaminoglycan bands. This result agrees well with the previous findings in that band far above 260 kDa was identified in the shells of crossed lamellar ultrastructure [15].

Previous studies have reported that homogeneous and crossed lamellar bivalve shells contain no noticeable protein bands on SDS-gels [16,19]. Negative staining might be attributed to the insufficient protein concentrations [38] as our recent works showed an evidence of shell-associated proteins in crossed lamellar [15] and homogeneous shell [7]. Organic biopolymer from homogeneous shells exhibited mesh-like proteinaceous network macromolecules [10] and acidic polysaccharides, for instance, glycosaminoglycans, more specifically proteoglycans [7]. Some of these components are challenging to stain — either diffuse, smear or stain faintly in SDS-electrophoresis [16,19,39]. While Alcian Blue stains a band far above 260 kDa (SI Fig. 3) and silver nitrate either reveals a negative band or stains faintly, combined staining techniques resulted in an increased sensitivity for glycoproteins and/or glycosaminoglycans. This is consistent with the observation that traditional protein stains such as Coomassie Blue and Silver nitrate bind feebly with proteoglycans and glycosaminoglycans in gels [15,40].

Primary residues of *the* shells studied here, comprising glycine, aspartate and proline are strikingly comparable to the amounts found in homogeneous *Arctica islandica* shell (39.9% in total vs 39.5%; SI Fig. 4). Further, solid state NMR spectra showed only low levels of biopolymers that cannot be assigned to any particular species, other than the interfacial carbonates for undecalcified *A. islandica* and *C. kingii* shells [10]. Although in the *C. kingii* shell, organic matrix extracts were below detectability. However, the insoluble organic extract of homogenous shell revealed features found previously for parchment and gelatin, denatured forms of collagen, and thus contains high proportions of glucosamine, galactosamine and galactose, an *O*-glycosylated sugar moiety that play a role in protein folding, interaction, and stability [41]. Galactose is not quantified in this study, but galactosamine after glucose is prominent and it therefore appears that the shell-associated organic matrix is more likely associated with the biochemistry of extracellular matrix protein and/or glycosylated fibrous proteins [33,42].

**Fig. 6:**
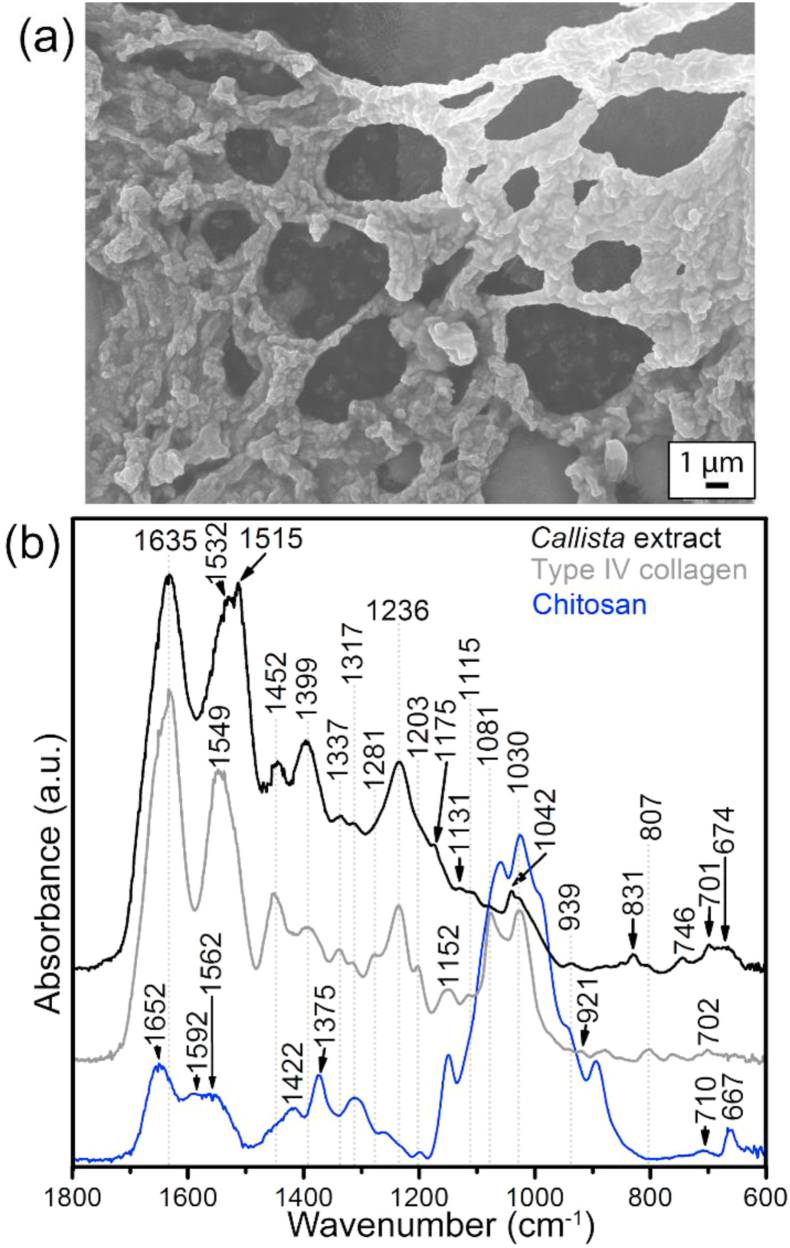
(a) Field emission gun scanning electron microscope image of representative unbleached insoluble acid extract from *Callista* shells. (b) FTIR spectrum of bleached insoluble acid extract from *Callista* shells (black), untreated type IV collagen (grey) and untreated chitosan (blue) for reference. The bands at ~1635 cm^−1^ and ~1337 cm^−1^ are assigned to the helical structure of matrix collagen and CH_2_ wagging of proline side chains. See Table 1 for assignments. (For interpretation of the references to colour in this figure legend, the reader is referred to the Web version of this article.)

The FEG-SEM imaging of the insoluble acid extract of *Callista* shells exhibits a morphological network matrix (Fig. 6a) as is typically found in homogeneous shell, but unlike the fibrous structure found in the nacreous shells [10]. The FTIR presents helical structures at 1635 cm^−1^ in amide I band, a 1337 cm^−1^ band has been used to identify collagen integrity (Fig. 6b) and is predominantly assigned to the CH_2_-wagging vibration of proline side chains of sequence structure of collagen matrix [28,32]. Also, the FTIR spectra of successive water-soluble organic and acid soluble moieties reveal characteristic amide III bands of collagen-related at ~1201 cm^−1^, ~1239 cm^−1^ and 1282 cm^−1^, and are mixed modes of CH_2_-wagging, C–N stretching and methyl deformation absorptions [28,30,32]. These vibrational modes are from glycine backbone and proline side chains. A 1031 cm^−1^ band is generally ascribed to the C–O vibration of hydroxyl groups (Table 1), either from glycosidic side chains or from hydroxylproline [30]. We could also identify the C–C stretching mode of proline at 857 cm^−1^ and ~931 cm^−1^. As reflected by the amino acid analysis as well as FTIR, *the* shells studied as compare to the homogenous shell are relatively high abundances in glycine, proline and polar amino acid, and appears compositionally closer to collagen-related matrix. Some forms of collagen matrix are found to be glycosylated with as much as a few percent of the amino acids and interact with glycosaminoglycans during fibril formation to influence the thickness of fibres [43], and the galactosamines are often found to be sulfonated [33]. Our FTIR and gel-SDS analyses reveal some bands that are attributed to the sulphated groups. Taken together, organic compositions of the crossed-lamellar and homogenous architectures in this study differ significantly from the nacreous ultrastructure that revealed silk-like amino acid compositions [10,11,19].

The structural model of organic biopolymers in shell biominerals includes hydrophobic amino acid side chains and sugar subunits [9,14]. Polysaccharide chitin is structurally cross-linked with the hydrophobic silk-like *β*-sheets in nacreous shells [[10], [11], [12]]. In contrast, the organic biomolecules in homogeneous shells constitute chitin and collagen-like matrix [10]. Generally, the evidence for polysaccharide chitin and its derivatives in bivalve shells is based majorly on histochemical staining (e.g. Refs. [[12], [13], [14]]) and on spectroscopic methods (e.g. Refs. [2,44,45]). More rarely, chromatographic separation [44] has been used to identify glucosamine, the monomer of chitin or chitosan, and demonstrated polysaccharide-chitin as a major and a common constituent in calcareous shells (e.g. Refs. [13,45]). In this study, the histochemical CFW dye for both *in*-situ and *ex*-situ follows the above mentioned results and depicts comparable polysaccharide-based biomolecules (Fig. 2c and d; Fig. 5c). This study also identified, by chromatographic separation, a relatively high abundance of glucosamine fraction (SI Fig. 2b) and secondary structure information from FTIR reveals prominent sugar-based bands. While chitin has been found in diverse organisms, recent studies revealed that this only occurs in layers in the shells molluscs such that thin organic fibre has a protein coat which is intimately associated with very minor *β*-chitin to produce a chitin-protein-complex fibre [10,12].

## 5 Conclusions

Although the total amount of shell macromolecules is low with about 1.38 wt% in *C. disrupta* and 1.71 wt% in *C. kingii*, our decalcification, purification, and staining protocols show discrete polysaccharide-based biomolecules and shell proteins. Our fixing and staining protocols reveal many discrete proteins and glycoproteins on the gel-SDS. The ‘singlet’ and ‘doublet’ bands far above 260 kDa are attributed to the glycosaminoglycans. Also some FTIR bands point to the sulphated glycosaminoglycans. The histochemical fixation and dye-staining technique of CFW on the gel-SDS exhibit deacetylated chitin (chitosan) from *Callista* shells for the first time. Our FTIR results of organic extracts reveal collagen-related amide bands in the bivalve venus shells and FEG-SEM imaging shows mesh-like collagenous network. As the structure-forming polysaccharides and proteins provide desired functional properties to a wide range of biomaterials, the organic biopolymer of *the* shells studied could be exploit further to discover new source of (collagenous) biomaterials.

## CRediT authorship contribution statement

**Oluwatoosin B.A. Agbaje:** Formal analysis, Writing - original draft, designed the study, carried out the analyses, interpreted the data and prepare the original manuscript. **J. Gabriel Dominguez:** Formal analysis, Writing - original draft, provided Callista shells and carried out optical microscopy analysis. **Dorrit E. Jacob:** Supervision, Writing - original draft, participated in the design of the study and supervised the study. All authors reviewed the manuscript and gave final approval for publication.

## Declaration of competing interest

The authors have no conflict to declare.
