# Supplementary material for: Organic biopolymers of venus clams: Collagen-related matrix in the bivalve shells with crossed-lamellar ultrastructure
Source: Biochem Biophys Rep. 2021 Feb 12;26:100939. doi: 10.1016/j.bbrep.2021.100939 (PMC7887640; doi:10.1016/j.bbrep.2021.100939)
Supplement: Multimedia component 1 [file mmc1.docx]

**Supplementary Information**

**Organic biopolymers of venus clams: collagen-related matrix in the bivalve shells with crossed-lamellar ultrastructure**

Oluwatoosin B. A. Agbaje^a,b,†,*^, J. Gabriel Dominguez^b^, Dorrit E. Jacob^a,τ^

^a^ Department of Earth and Environmental Sciences, Macquarie University, Sydney, Australia.

^b^ Department of Biological Sciences, Macquarie University, Sydney, Australia.

^†^ Current Address: Department of Earth Sciences, Palaeobiology, Uppsala University, Uppsala, Uppsala, Sweden

τ Current Address: Research School of Earth Sciences, Australian National University, Canberra, Australia

*** Correspondence:** toosin.agbaje@mq.edu.au; [toosin91014@gmail.com](mailto:toosin91014@gmail.com)


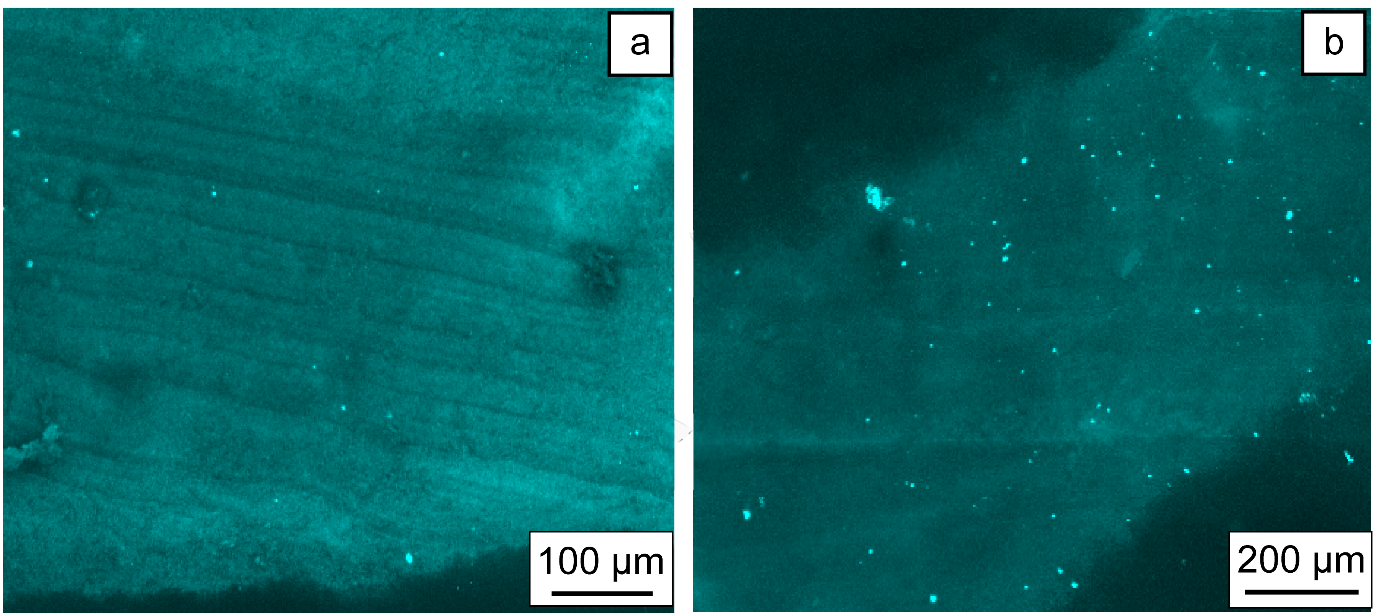


**SI Figure 1.** (a) Etched section of Callista shell showing the presence of polysaccharide-based biopolymers in the growth lines in the inner shell layer after staining with Calcofluor White M2R. (b) Fluorescence image of the unstained decalcified *Callista* shell


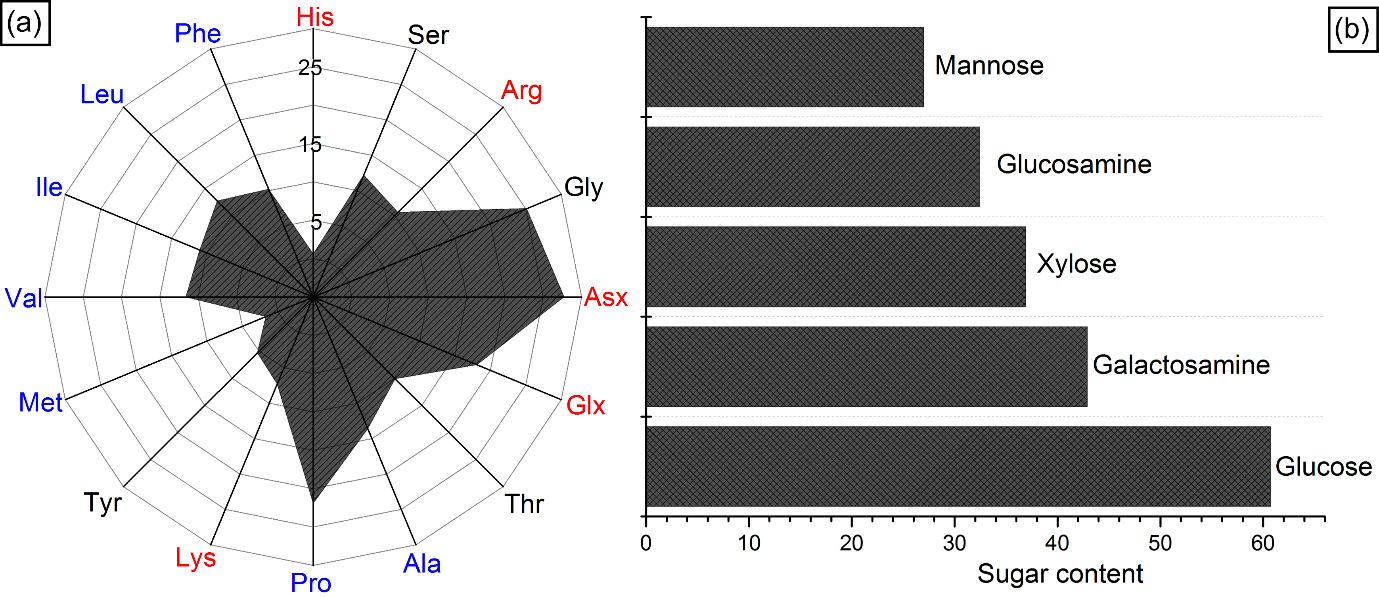


**SI Figure 2.** Total amino acid compositions of *Callista* shells and proportions from combining the water-soluble (soluble organic moiety) plus the Trichloroacetic acid-Phosphate buffer soluble moieties (acid soluble moiety) (a); Total monosaccharide compositions of *Callista* shells and proportions taken together from the water-soluble (SOM) plus the Trichloroacetic acid-Phosphate buffer soluble moieties (acid soluble moiety) (b). NB: The compositions involved the percentage of two fractions, i.e. water-soluble organic moiety and acid soluble moiety.


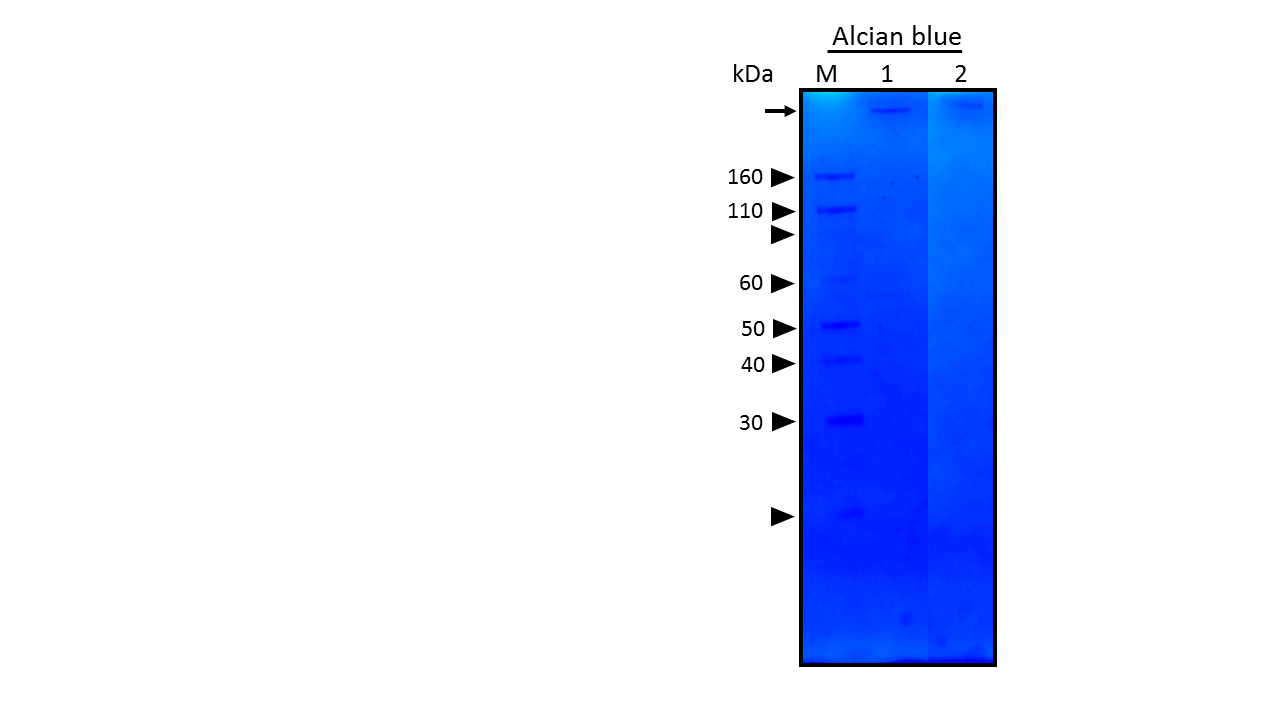


**SI Figure 3.** SDS-PAGE of water-soluble organic moiety (lane 1) and acid soluble moiety (Lane 2) (lane 2). Lane M: Molecular weight (Novex Sharp Pre-stained Protein; 5 µL) standards with masses in kDa are indicated. Samples were stained with only Alcian Blue.


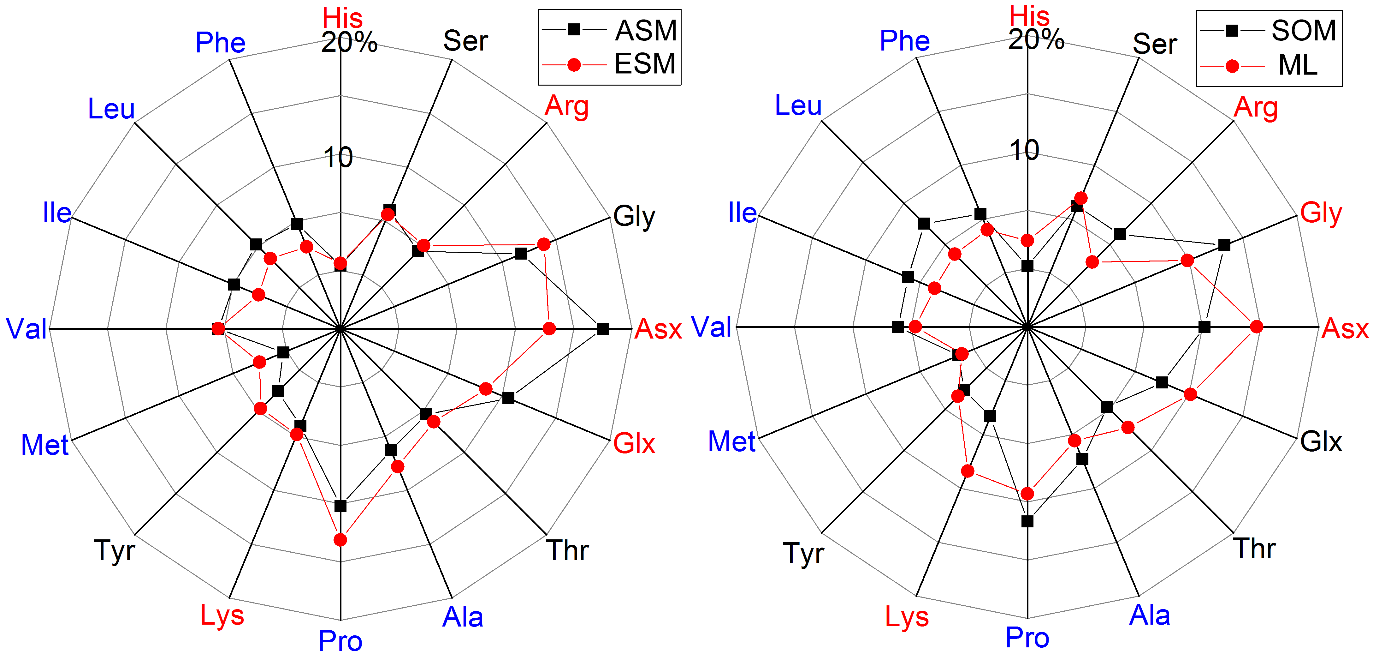


**SI Figure 4.** Amino acid compositions of Trichloroacetic acid-Phosphate buffer soluble moieties (Acid Soluble Moiety; **ASM**) and Water-Soluble Organic Matrix **(SOM)**, comparing with the EDTA-Soluble Organic Matrix **(ESM)** in shells of homogeneous *A. islandica* from Agbaje et al.(Agbaje et al. 2017) and *Mercenaria stimpsoni* **(MS)** soluble organic matrix from Samata (Samata 1990).

**References**

O.B.A. Agbaje, D.E. Thomas, B.V. Mclnerney, M.P. Molloy, D.E. Jacob, Organic macromolecules in shells of *Arctica islandica*: comparison with nacroprismatic bivalve shells, Mar. Biol. 164 (2017) 208, https://doi.org/10.1007/s00227-017-3238-2

T. Samata, Ca-binding glycoproteins in molluscan shells with different types of ultrastructure, Veliger 33(2) (1990) 190-201.
